# Enhanced Interfacial Binding and Electron Extraction Using Boron‐Doped TiO_2_ for Highly Efficient Hysteresis‐Free Perovskite Solar Cells

**DOI:** 10.1002/advs.201901213

**Published:** 2019-09-10

**Authors:** Xiaoqiang Shi, Yong Ding, Shijie Zhou, Bing Zhang, Molang Cai, Jianxi Yao, Linhua Hu, Jihuai Wu, Songyuan Dai, Mohammad Khaja Nazeeruddin

**Affiliations:** ^1^ State Key Laboratory of Alternate Electrical Power System with Renewable Energy Sources North China Electric Power University Beijing 102206 P. R. China; ^2^ Key Laboratory of Photovoltaic and Energy Conservation Materials Institute of Applied Technology Hefei Institutes of Physical Science Chinese Academy of Sciences Hefei 230031 Anhui P. R. China; ^3^ Fujian Provincial Key Laboratory of Photoelectric Functional Materials Institute of Materials Physical Chemistry Huaqiao University Xiamen 361021 P. R. China; ^4^ Group for Molecular Engineering of Functional Materials Institute of Chemical Sciences and Engineering École Polytechnique Fédérale de Lausanne CH‐1951 Sion Switzerland

**Keywords:** charge transport, hysteresis, interfacial binding, perovskite solar cells, titanium dioxide

## Abstract

Perovskite solar cells (PSCs) have witnessed astonishing improvement in power conversion efficiency (PCE), more recently, with advances in long‐term stability and scalable fabrication. However, the presence of an anomalous hysteresis behavior in the current density–voltage characteristic of these devices remains a key obstacle on the road to commercialization. Herein, sol–gel‐processed mesoporous boron‐doped TiO_2_ (B‐TiO_2_) is demonstrated as an improved electron transport layer (ETL) for PSCs for the reduction of hysteresis. The incorporation of boron dopant in TiO_2_ ETL not only reduces the hysteresis behavior but also improves PCE of the perovskite device. The simultaneous improvements are mainly ascribed to the following two reasons. First, the substitution of under‐coordinated titanium atom by boron species effectively passivates oxygen vacancy defects in the TiO_2_ ETL, leading to increased electron mobility and conductivity, thereby greatly facilitating electron transport. Second, the boron dopant upshifts the conduction band edge of TiO_2_, resulting in more efficient electron extraction with suppressed charge recombination. Consequently, a methylammonium lead iodide (MAPbI_3_) photovoltaic device based on B‐TiO_2_ ETL achieves a higher efficiency of 20.51% than the 19.06% of the pure TiO_2_ ETL based device, and the hysteresis is reduced from 0.13% to 0.01% with the B‐TiO_2_ based device showing negligible hysteresis behavior.

## Graphical abstract

**Mesoporous boron‐doped TiO_2_ (B‐TiO_2_)** is demonstrated as an improved electron transport layer (ETL) for perovskite solar cells for the reduction of hysteresis. The incorporation of boron dopant in TiO_2_ ETL not only reduces the hysteresis but also improves device performance. Consequently, a methylammonium lead iodide photovoltaic device based on B‐TiO_2_ ETL achieves a promising efficiency of 20.51% with negligible hysteresis.

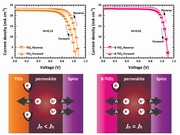

## 1 Introduction

Since the first demonstration of perovskite solar cells (PSCs) by Miyasaka and co‐workers in 2009, tremendous attention has been devoted to this field due to their rapidly increased power conversion efficiency (PCE) and potentially low fabrication cost.^1^ In the past few years, the PCE of PSCs achieved an astonishing improvement from 3.8% up to 24.2%.^2^ However, one of the most critical challenges facing the PSC community is the current density–voltage (*J*–*V*) hysteresis behavior, which means that the *J*–*V* scans yield different results depending on the scan direction and scan rate.^3^ It has been considered that hysteresis is caused by the presence of both mobile ionic species and trap assisted and/or surface charge recombination.^4^ While the dominant factors affecting hysteresis are being debated within the community, one source that must be considered is the electron transport layer (ETL), particularly when it is composed of mesoporous titanium dioxide (TiO_2_) nanoparticles.[qv: 3b] TiO_2_ is susceptible to intrinsic defects, which mainly include oxygen vacancies, cation vacancies, and cation interstitials.^5^ These nonstoichiometry defects can be ionized to form undesirable electronic defects (quasi‐free electrons and electron holes) and result in reduced charge transport.^6^ As a result, PSCs based on TiO_2_ with these defects suffer from strong charge accumulation and a low photovoltage. This encourages more effort to explore efficient ETLs with higher conductivity and less defects.^7^

In order to improve the charge transport properties of TiO_2_ ETLs, surface modifications and doping strategies have been developed.^8^ Tan et al. reported a contact passivation strategy using chlorine as the capping ligand to modify the low‐temperature‐processed TiO_2_ ETL to mitigate interfacial recombination and improve the interface binding in planar PSCs. They got hysteresis‐free planar PSCs with enhanced efficiency and stability.^9^ In addition, Hu et al. synthesized amino‐functionalized TiO_2_ nanoparticles, and achieved high efficiency with suppressed hysteresis by applying NH_2_‐TiO_2_ as the ETL.^10^ Doping is another effective approach to modify both the electronic band structures and trap states of TiO_2_. To tackle this problem, a variety of dopants, such as Al, Y, Nb, and Li, have been investigated to manipulate carrier behavior.^11^ Figure S13 in the Supporting Information shows plots of the hysteresis index (HI) against dopant species for PSCs reported so far, and the detailed performance parameters are listed in Table S2 in the Supporting Information. Li dopant exhibits significant performance change in terms of reduced hysteresis, but the open‐circuit voltage (*V*_oc_) of PSCs is also reduced due to the downward shift of conduction band minimum (CBM) of TiO_2._[qv: 11d,12] It should be noted that downshift of TiO_2_ CBM does not always cause *V*_oc_ drop as it is not the only factor affecting the voltage. *V*_oc_ can be enhanced or maintained when these dopants passivate electronic trap sites in the bulk and at the surface of the TiO_2_.[qv: 11d,13] By comparison, dopants such as Nb that upshift the CBM of TiO_2_ should be more desirable. Nevertheless, the hysteresis effect has not been resolved well for these dopants (Table S2 and Figure S13, Supporting Information). Doping in the TiO_2_ lattice can produce considerable lattice disorder and defects, thus causing the decrease of electron mobility and the increase of parasitic recombination.^14^ Considering the theoretical model of the electrical conductivity, the ideal state is to have both high electron concentration and high electron mobility in the TiO_2_ ETL simultaneously to achieve efficient electron extraction and hence balanced carrier transport. Previously, we have shown that boron (B) doping could enhance the electron injection from excited dye molecules to TiO_2_ photoanode, resulting in the increased PCE of dye‐sensitized solar cells. Recently, Tian et al. reported that a low concentration of boron doping could enhance the carrier mobility and electrical conductivity of TiO_2_.^15^ Therefore, B‐doped TiO_2_ (abbreviated as B‐TiO_2_) is expected to be a desirable ETL for PSCs to synchronously improve the PCE and significantly reduce the hysteresis.

Herein, we demonstrate sol–gel‐processed mesoporous B‐TiO_2_ as an improved ETL for PSCs. B‐TiO_2_ ETL exhibits upshifted CBM, increased electron mobility and conductivity, and reduced electron traps. Our density functional theory (DFT) results show that B doping leads to stronger binding at the ETL/perovskite interface with a total binding energy of −5.41 eV for TiO_2_/perovskite interface and −6.63 eV for the B‐TiO_2_/perovskite interface. These calculation results are further supported by steady‐state photoluminescence (PL), time‐resolved photoluminescence (TRPL), and thermal admittance spectroscopy (TAS) results. The incorporation of B dopant in TiO_2_ ETL not only reduces the hysteresis behavior but also improves *V*_oc_, short‐circuit current density (*J*_sc_), and fill factor (FF) of perovskite device. As a result, PSC based on B‐TiO_2_ ETL demonstrates a higher efficiency of 20.51% than 19.06% of an undoped TiO_2_ ETL based device, and the hysteresis is reduced from 0.13% to 0.01% with the B‐TiO_2_ based device showing negligible hysteresis behavior.

## 2 Results and Discussion

### 2.1 Incorporation of Boron Dopant in TiO_2_ Nanoparticles

Pure TiO_2_ and B‐TiO_2_ nanoparticles were synthesized by a facile sol–gel method and subsequent hydrothermal treatment, as schematically illustrated in Figure S1a in the Supporting Information.^16^ In this approach, boric acid as the dopant source was added into the precursor solution to achieve a substitution doping level of 2%.[qv: 15,16b] Transmission electron microscopy (TEM) analysis revealed that the TiO_2_ and B‐TiO_2_ (Figure S1b,c, Supporting Information) samples are well‐dispersed nanoparticles, and both have a narrow distribution in the average size of ≈21 nm (Figure S2, Supporting Information). The B‐TiO_2_ sample (Figure S1d, Supporting Information) shows an unchanged interplanar spacing of 0.358 nm for the (101) plane of anatase TiO_2_. Scanning electron microscopy (SEM) images of both TiO_2_ and B‐TiO_2_ (Figure S1e,f, Supporting Information) films exhibit typical porous surface structure, which will facilitate the infiltration of perovskite precursor solution. Furthermore, atomic force microscopy (AFM) and corresponding cross‐section SEM views show that B doping leads to a slight decrease of the root‐mean‐square (RMS) roughness compared to pristine TiO_2_ (Figure S3, Supporting Information).

X‐ray diffraction (XRD) patterns (Figure S4a, Supporting Information) confirm that a pure anatase phase (JCPDS No. 21‐1272) formed after calcination and there was no observable structural difference between TiO_2_ and B‐TiO_2_ samples. The peak positions (Figure S4b, Supporting Information) of B‐TiO_2_ did not show any discernible difference to pure anatase, and the values of the full width at half maximum (FWHM) of these two samples were almost the same (0.522 for TiO_2_ and 0.524 for B‐TiO_2_). To confirm this, XRD patterns were measured of TiO_2_ and B‐TiO_2_ powder scraped from corresponding sintered films and XRD patterns of TiO_2_ and B‐TiO_2_ films deposited on fluorine‐doped tin oxide (FTO)/cp‐TiO_2_ are also presented (Figure S4c,d, Supporting Information). It can be inferred that the doping level of 2% had insignificant effect on the crystal structure of TiO_2_.

**Figure 1:**
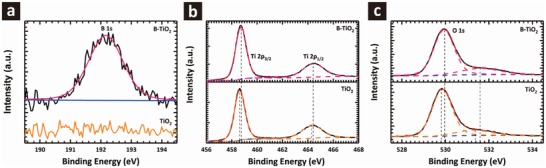
a) XPS spectra of B 1s peak. b) XPS spectra of Ti 2p peaks. c) XPS spectra of O 1s peaks.

To verify the form of incorporation of B in the TiO_2_ lattice, X‐ray photoelectron spectroscopy (XPS) measurements were conducted. **Figure** **1**a–c compares the XPS spectra of the B 1s, Ti 2p, and O 1s peaks for doped and undoped samples, respectively. Figure 1a shows the XPS spectrum of B 1s for the B‐TiO_2_ sample, with a single peak centered at about 192.1 eV, which is consistent with literature values.^17^ As expected, the TiO_2_ counterpart does not have a corresponding peak. Figure 1b shows the Ti 2p_1/2_ and Ti 2p_3/2_ peaks of the TiO_2_ sample at binding energies of ≈464.3 and ≈458.6 eV, respectively, which are typical for the Ti—O bonds in TiO_2._ 18 In the case of B‐TiO_2_, the Ti 2p_3/2_ peak is shifted slightly higher to 458.7 eV, resulting from charge transfer through B doping, suggesting that the B dopant was incorporated in the TiO_2_ lattice without the formation of a separate phase. In Figure 1c, the major component of the O 1s peaks centered at 529.9 and 530.0 eV can be ascribed to bonding with Ti and B, while the other component near the binding energies of 531.3 and 531.6 eV is generally considered to be hydrated bonds or carbonate from surface contaminations. The slightly higher O 1s binding energy for the B‐TiO_2_ sample further suggests the linkage of the Ti—O—B bond in B‐TiO_2_. Moreover, based on the XPS fitting results, the amount of B in the B‐TiO_2_ sample is evaluated by an elemental sensitive factor method,^19^ which reveals that the actual B doping level is 2.18 at%.

### 2.2 Optical and Electrical Properties of B‐TiO_2_ Film

**Figure 2:**
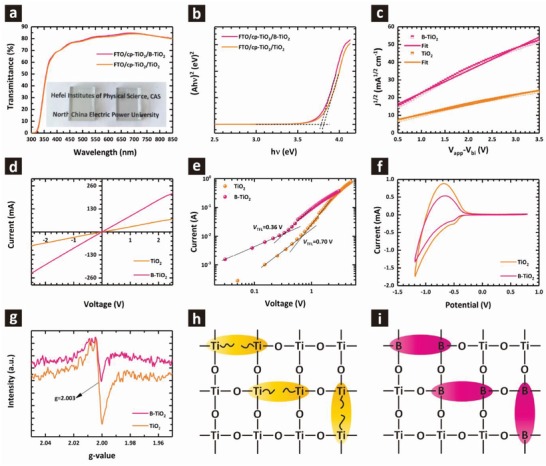
a) Transmittance spectra of the TiO_2_ and B‐TiO_2_ films. The inset shows the corresponding photographs of both the films. b) Extrapolated plots (*A*hν)^2^ versus hν obtained from the absorption spectra of TiO_2_ and B‐TiO_2_ films. c) Electron mobility of TiO_2_ and B‐TiO_2_ films. d) *I*–*V* characteristics of the FTO/cp‐TiO_2_/TiO_2_/Ag and FTO/cp‐TiO_2_/B‐TiO_2_/Ag devices. e) Log–log plot of *I*–*V* curves of TiO_2_ and B‐TiO_2_ devices revealing *V*_TFL_ tuning point behavior. f) Cyclic voltammetry of TiO_2_ and B‐TiO_2_ films in LiClO_4_ (0.1 m) solution. g) Electron paramagnetic resonance (EPR) spectra at 5 K for TiO_2_ and B‐TiO_2_ powder. h) Oxygen vacancies in the lattice give rise to Ti defects that form deep electronic traps. i) Boron substitution in the Ti sites passivates these defects.

High optical transmittance of the charge transport layer is crucial for maximizing the photocurrent of perovskite devices. **Figure** **2**a compares the transmission spectra of FTO glass covered with the TiO_2_ and B‐TiO_2_ films. Both of them display high average transmittance in the visible region, demonstrating excellent optical quality. The UV–vis absorption spectra in Figure S5 in the Supporting Information show a slight red shift with B doping. Figure 2b shows the extrapolated plots of (*A*hν)^2^ versus hν, which were obtained from the absorption spectra of TiO_2_ and B‐TiO_2_ samples. The bandgap (denoted by *E*_g_) of the TiO_2_ is 3.81 eV while that of B‐TiO_2_ is 3.78 eV, which is consistent with that reported in the literature.^20^

Ultraviolet photoelectron spectroscopy (UPS) measurements were also performed to ascertain if there is any change of the energy levels of the TiO_2_ films before and after doping. Figure S6a,b in the Supporting Information shows that the secondary electron cutoffs of both TiO_2_ and B‐TiO_2_ is 16.14 eV corresponding to the work function of ≈5.06 eV. Figure S6c in the Supporting Information shows valence band spectra, from which the energy differences between the valance band (denoted by *E*_v_) edges and Fermi level (denoted by *E*_f_) of TiO_2_ and B‐TiO_2_ were found to be 2.84 and 2.78 eV, respectively. The *E*_v_ of TiO_2_ and B‐TiO_2_ was evaluated to be −7.90 and −7.84 eV, respectively. Finally, combining with optical bandgaps, the conduction band (denoted by *E*_c_) edges of TiO_2_ and B‐TiO_2_ were determined to be −4.09 and −4.06 eV, respectively. In Figure S6d in the Supporting Information, we illustrate that the *E*_c_ of B‐TiO_2_ is shifted by around 0.03 eV toward the vacuum level, which is beneficial for enhancing *V*_oc_.[qv: 14b]

In addition to energy band alignment, the carrier mobility also affects the charge transfer process. The electron mobilities of TiO_2_ and B‐TiO_2_ samples were measured by the space charge limited current (SCLC) model employing an electron‐only device configuration (Figure S7b, Supporting Information). The detailed calculation process is provided in Note S1 in the Supporting Information. Figure 2c shows current density–voltage (*J*–*V*) curves of the electron‐only device fitting the Mott–Gurney law.^21^ The calculated electron mobility of the TiO_2_ is 3.30 × 10^−5^ cm^2^ V^−1^ s^−1^, while it increases to 1.69 × 10^−4^ cm^2^ V^−1^ s^−1^ after B doping, about five times higher than the former. The higher electron mobility would contribute significantly to the electron transport, and therefore higher *J*_sc_ and FF in the perovskite cells. To determine direct current conductivity (σ_0_) of TiO_2_ and B‐TiO_2_ samples, we fabricated FTO/cp‐TiO_2_/TiO_2_ or B‐TiO_2_/Ag devices, and the current–voltage (*I*–*V*) characteristics are shown in Figure 2d. The conductivity can be obtained from the slope of the *I*–*V* plot, *I* = σ_0_ *AD* ^−1^ V, where *A* is the sample area (9 mm^2^) and *D* is the film thickness (180 nm). The calculated values of the conductivity are 6.00 × 10^−3^ and 1.81 × 10^−2^ mS cm^−1^ for TiO_2_ and B‐TiO_2_, respectively, which is consistent with the variation trend of the electron mobility (Table S1, Supporting Information).

Low mobility of the ETLs indicates more trap states, which would lead to charge accumulation at the interface and poor charge transport. To investigate the effects of B doping on the electron trap state density in the TiO_2_ films, the dark *I*–*V* curves of TiO_2_ and B‐TiO_2_ devices (Figure S7a, Supporting Information) were measured, as shown in Figure 2e and Figure S14 in the Supporting Information. Obviously, the linear relationship indicates an ohmic contact at low bias voltage region, while a nonlinearity increase starts to appear when the bias voltage exceeds the inflexion point, demonstrating that the trap states are completely filled. The trap‐filled limit voltage (*V*_TFL_) was determined by the trap state density^22^

(1) $$V_{\mathrm{TFL}}=\frac{en_{t}L^{2}}{2\varepsilon_{0}\varepsilon_{r}}$$

where *e* is the elementary charge of the electron (1.6 × 10^−19^ C), *L* is the TiO_2_ film thickness (180 nm), ε_0_ is the vacuum permittivity (8.854 × 10^−12^ F m^−1^), ε_r_ is the relative dielectric constant of TiO_2_ (55),^23^ and *n*_t_ is the trap state density. With the averaged *V*_TFL_ value (Figure S14, Supporting Information), the trap state density *n*_t_ in B‐TiO_2_ was calculated to be 6.39 × 10^16^ cm^−3^. For comparison, the calculated trap state density in the TiO_2_ was 1.27 × 10^17^ cm^−3^, which is almost two times higher than in the B‐TiO_2_. Clearly, B doping effectively passivates electron traps, resulting in the increased electron mobility.

To further understand the surface states of TiO_2_ films, we conducted cyclic voltammetry (CV) analysis in LiClO_4_ (0.1 m) solution. Under negative potential (forward bias), the quasi *E*_f_ of TiO_2_ moved toward to *E*_c_, leading to a capacitive current. Ideally, electron injection will commence once the quasi *E*_f_ approaches the *E*_c_ minimum. However, electrons originating from surface states level below the *E*_c_ are injected under forward bias, producing a gradual onset of the capacitive current. Under reverse bias, the capacitive current reached zero at the more positive potentials, implying recovery of the injected electrons and regeneration of most surface states. To sum up, the current peak is related to the occupancy of the surface states in the bandgap.^24^ As shown in Figure 2f, the current peak intensity of B‐TiO_2_ sample decreased compared to that of TiO_2_. This further confirms the reduced electron traps in the presence of B doping.

There is broad consensus that oxygen vacancies or titanium interstitials are the predominant nonstoichiometry defects in TiO_2_, resulting partially from the reduction of Ti^4+^ to Ti^3+^ (treating the electronic structure as purely ionic while the actual electronic structure is part covalent). As a result, the inferred Ti^3+^ defects can induce a shallow energy level below the *E*_c_ and act as electron trap sites, which traps electrons and depresses electron transport and conductivity of TiO_2_. Based on the above measurements and discussion, we show the trap passivation mechanism in Figure 2h,i. We speculate that the process of B doping is similar to that of Al doping, both of which involve the substitution of the inferred Ti^3+^ by a species with valency +3.[qv: 11a] Therefore, this substitution by B effectively passivates oxygen vacancy defects in a TiO_2_ ETL. This conclusion is further supported by the electron paramagnetic resonance (EPR) results as indicated in Figure 2g, in which signal originating from oxygen vacancy traps (*g* = 2.003) is significantly weakened.^25^

### 2.3 Enhanced Interfacial Binding and Electron Extraction

**Figure 3:**
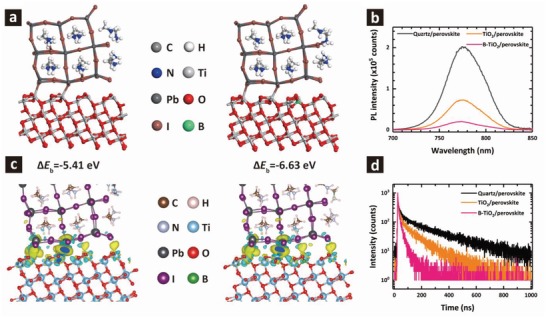
a,c) Binding energy and corresponding differential charge densities of the two structures. b,d) Steady‐state PL spectra and TRPL decays of perovskite films on quartz and on TiO_2_ and B‐TiO_2_ coated FTO substrates.

Considering B‐TiO_2_ film with reduced defects, the ETL/perovskite interface can be expected to have higher binding energy, that is, be more ideal. To confirm this, we first used DFT to examine interfacial binding at the ETL/perovskite interface (**Figure** **3**a,c). Details for DFT calculations is provided in Note S2 in the Supporting Information. Boron doping leads to stronger binding at the ETL/perovskite interface with a total binding energy of −5.41 eV for TiO_2_/perovskite interface and −6.63 eV for the B‐TiO_2_/perovskite interface. It indicates that B‐TiO_2_ film surface is more energetically favored to contact the perovskite. We speculated that the increased binding energy at B‐TiO_2_/perovskite interface might originate from the stronger B—I bonds.^26^ Besides, B‐TiO_2_/perovskite interface shows stronger Coulomb interactions as confirmed by the differential charge density across this interface.

Steady‐state photoluminescence measurement was performed to study the charge transfer kinetics between perovskite and ETLs. Figure 3b comparatively shows steady‐state PL spectra of methylammonium lead iodide (MAPbI_3_) films where we observe a more strongly quenched PL in the B‐TiO_2_/perovskite stack. This result suggests that the electron extraction from the perovskite was more efficient for B‐TiO_2_, consistent with the high electron mobility of B‐TiO_2_ film and improved interfacial binding at the B‐TiO_2_/perovskite interface. We further investigate the modulation of charge transfer through time‐resolved photoluminescence decay kinetics (Figure 3d). The TRPL decay curves were exponentially fitted with the lifetime and corresponding amplitudes listed in **Table** **1**. Generally, the slow decay component (τ_2_) can be correlated to the radiative recombination of free carriers due to bulk traps in the perovskite, and the fast decay component (τ_1_) is primarily dictated by the quenching of carriers at the interface, in the present case involving the ETL/perovskite interface.^9^, ^12^ As shown in Table 1, the time constant for fast decay component (τ_1_) decreases from 8.2 to 4.7 ns for perovskite cast on B‐TiO_2_ film, indicative of a more rapid interfacial charge transfer in the B‐TiO_2_/perovskite stack. This result originates from the enhanced electron mobility and improved interface. Meanwhile, the slow decay component (τ_2_) decreases from 116.8 to 27.0 ns, which may result from a reduced Shockley–Read–Hall recombination via bulk traps in the perovskite film, given the improved film quality with B‐TiO_2_ ETL (Figure S8, Supporting Information).^27^ Compared to the TiO_2_/perovskite sample, the calculated amplitude of τ_2_ is decreased from 72.0% to 64.6%, while the calculated amplitude of τ_1_ is increased from 28.0% to 35.4%, resulting in amplitude average lifetime of 86.4 and 19.1 ns for TiO_2_ and B‐TiO_2_ based stack, respectively. Overall, these results further indicate that the interfacial binding and electron extraction are enhanced at B‐TiO_2_/perovskite interface. In addition, perovskite deposited on quartz substrate shows high PL intensity and long carrier lifetime, indicating the good quality of our perovskite film.

**Table 1:** TRPL decay lifetimes of perovskite films on quartz, TiO_2_, and B‐TiO_2_ coated FTO substrates. τ_1_ and τ_2_ correspond to the fast and slow decay components, respectively

Samples	τ_1_ [ns]	*A*_1_ [%]	τ_2_ [ns]	*A*_2_ [%]	τ_ave_ [ns]^a)^
Quartz/perovskite	15.3	12.6	241.0	87.4	212.6
TiO_2_/perovskite	8.2	28.0	116.8	72.0	86.4
B‐TiO_2_/perovskite	4.7	35.4	27.0	64.6	19.1

^a)^ $\tau_{\mathrm{ave}}=\sum_{i}A_{i}\tau_{i},\;\mathrm{where}\;\sum_{i}A_{i}\;=\;1.\;\;$

### 2.4 Photovoltaic Performance of PSCs

**Figure 4:**
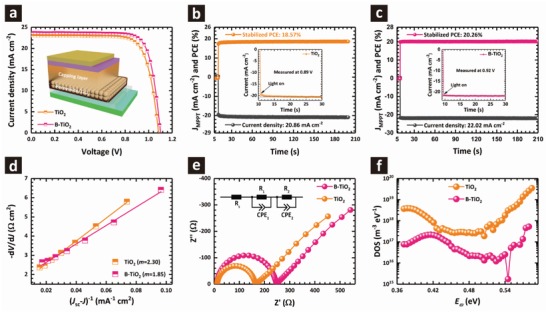
a) *J*–*V* curves of the devices with TiO_2_ and B‐TiO_2_ ETLs. b,c) Steady‐state power output at the maximum power point under ambient conditions (20 to 25 °C; relative humidity of 25–40%). The insets are zoomed‐in view. d) Plots of −d*V*/d*J* versus (*J*_sc_ − *J*)^−1^ for the typical devices and corresponding linear fitting results. e) Nyquist plots of the devices with TiO_2_ and B‐TiO_2_ ETLs obtained at 0.8 V bias under illumination (inset shows the equivalent circuit for fitting the Nyquist plots). f) Trap density of states obtained by thermal admittance spectroscopy for devices with TiO_2_ and B‐TiO_2_ ETLs.

With the superior optoelectronic properties discussed above, it is expected that the B‐TiO_2_ would make a better ETL in the PSCs than the TiO_2_. Mesoscopic‐type PSCs were therefore fabricated based on different ETLs with the device structure shown in **Figure** **4**a. The simple and commonly used MAPbI_3_ was employed as the active absorber.

Figure 4a shows the *J*–*V* characteristics of mesoscopic‐type PSCs using different ETLs, with the photovoltaic parameters, including *J*_sc_, *V*_oc_, FF, and PCE summarized in **Table** **2**. The device based on TiO_2_ produces a calculated PCE of 19.06% with *J*_sc_ = 22.96 mA cm^−2^, *V*_oc_ = 1.08 V, and FF = 76.60%. As expected, when the B‐TiO_2_ is employed as ETL, the *J*_sc_, *V*_oc_, and FF are increased to 23.71 mA cm^−2^, 1.10 V, and 78.60%, yielding a PCE up to 20.51%. The statistical consistency of the performance enhancement was demonstrated by comparing the performance of 24 devices (24 devices each for pure TiO_2_ and B‐TiO_2_), and the results are shown in Figure S9 in the Supporting Information. The low device performance for the TiO_2_ based PSCs is caused by relatively small *J*_sc_ and FF, which is related to low electron mobility and higher resistance, and the low *V*_oc_ results from the carrier recombination at the ETL/perovskite interface (see later discussion). By comparison, the mesoscopic‐type PSCs with B‐TiO_2_ ETLs exhibit better performance. The higher *J*_sc_ and FF are attributed to the high electron mobility that promotes effective electron extraction, and the larger *V*_oc_ is due to the stronger bonds at the B‐TiO_2_/perovskite interface that results in reduced interfacial recombination. Figure S10 in the Supporting Information shows the incident photon to current conversion efficiency (IPCE) of the PSCs based on different ETLs. Integration of the IPCE spectra over the solar emission yields AM 1.5 photocurrent of 21.93 and 22.60 mA cm^−2^ for the device using TiO_2_ and B‐TiO_2_, respectively, which agrees well with the *J*_sc_ values obtained from the *J*–*V* curves within ≈5% deviation. It can be seen that the integrated current density is lower than what is measured from the *J*–*V* curves with and without boron doping. This trend has been systematically observed also from other groups, and it can in part be attributed to a small spectral mismatch between the solar simulator and the standard AM 1.5G emission.^28^

**Table 2:** Photovoltaic parameters of MAPbI_3_ solar cells based on TiO_2_ and B‐TiO_2_ ETLs

Devices	Scan direction	*J*_sc_ [mA cm^−2^]	*J*_sc_ by IPCE [mA cm^−2^]	*V*_oc_ [V]	FF [%]	PCE [%]	HI	Stabilized [%]
TiO_2_	Reverse	22.96	21.93	1.08	76.60	19.06	0.13	18.57
	Forward	22.81		1.03	70.17	16.57		
B‐TiO_2_	Reverse	23.71	22.60	1.10	78.60	20.51	0.01	20.26
	Forward	23.57		1.10	78.13	20.34		

To further demonstrate the device characteristics, photocurrent response of the champion devices from each group based on TiO_2_ and B‐TiO_2_ was measured when the devices were biased at 0.89 and 0.92 V, respectively. Figure 4b,c shows the corresponding curves at the maximum power point in the *J*–*V* plots. The PCEs of the champion devices using the TiO_2_ and B‐TiO_2_ ETLs stabilize at 18.57% and 20.26% with photocurrent densities of 20.86 and 22.02 mA cm^−2^, respectively, very close to the values measured from the *J*–*V* curves. As shown in Figure 4b,c insets, the photocurrent of the B‐TiO_2_ based device rose quickly to maximum steady‐state photocurrent. However, photocurrent from the TiO_2_ based cell needed more than 2 s to fully saturate. It indicates that it takes longer for the TiO_2_ based device to reach steady state, which is consistent with the larger hysteresis of TiO_2_ based devices (Table 2).^29^

To gain further insight into the photovoltaic characteristics of TiO_2_ and B‐TiO_2_ based devices, we analyzed the *J*–*V* characteristics based on the equivalent circuit model (Figure S12, Supporting Information). According to the equivalent circuit model, the current density *J* is described as^30^

(2) $$J=J_{\mathrm{ph}}-J_{0}\left[\exp\left(\frac{q\left(V+J\times R_{s}\right)}{mk_{B}T}\right)-1\right]-\frac{\;V+J\times R_{s}}{R_{\mathrm{sh}}}$$

where *J*_ph_ is the photocurrent density, *J*_0_ is the saturation current density at reverse bias, *q* is the elementary charge, *m* is the ideality factor, *k*_B_ is the Boltzmann's constant, *T* is temperature in degrees Kelvin, *R*_s_ is the series resistance, and *R*_sh_ is the shunt resistance. When *R*_s_ is far less than *R*_sh_, Equation (2) can be expressed as

(3) $$-\frac{dV}{dJ}\;=\;\frac{mk_{B}T}{q}{\left(J_{\mathrm{sc}}\;-\;J\right)}^{-1}\;+\;R_{s}$$

Figure 4d displays the plots of −d*V*/d*J* versus (*J*_sc_ − *J*)^−1^ and the linear fitting curves for TiO_2_ and B‐TiO_2_ based devices according to Equation (3). The values of *m* can be derived from the linear fitting results. Compared to the TiO_2_ based device, the value of *m* decreased from 2.30 to 1.85. The ideality factor *m* depends on carrier diffusion and recombination processes.[qv: 30a] The smaller the value of *m*, the less recombination is caused by traps in the device. Thus, a smaller *m* perhaps means that the carrier recombination in B‐TiO_2_ based device is reduced because of the reduction of trap states in the B‐TiO_2_ films.

The electrical impedance spectroscopy (EIS) measurements were conducted to extract transfer resistance in the devices. Figure 4e shows the Nyquist plots of the devices using TiO_2_ and B‐TiO_2_ ETLs measured at 0.8 V bias under illumination, with the equivalent circuit shown in Figure 4e inset. Commonly, the high‐frequency component is the signature of the transfer resistance *R*_tra_ (*R*_1_) and the low‐frequency component is for the recombination resistance *R*_rec_ (*R*_2_).^31^ In this study, because other interfaces are identical for all devices, the only variable affecting *R*_tra_ is the perovskite/ETL interface. When we perform numerical fitting, the device with B‐TiO_2_ ETL shows smaller *R*_tra_ of 6.45 Ω and the larger *R*_rec_ of 231.4 Ω compared to that of TiO_2_ based device (*R*_tra_ of 8.33 Ω and *R*_rec_ of 147.2 Ω). The smaller *R*_tra_ is beneficial for electron extraction, and the large *R*_rec_ effectively resists charge recombination, which is in agreement with the observations discussed above.

To further investigate the performance improvement by using B‐TiO_2_ ETL, we studied the density of trap states at the perovskite/ETL interface by performing thermal admittance spectroscopy on complete devices. By interrogating the frequency dependent capacitance via TAS measurements we can determine the energetic profile of trap density of state (DOS). As shown in Figure 4f, the B‐TiO_2_ based device shows more than one order of magnitude lower trap density compared to the control device, confirming that the electronic contact between perovskite and TiO_2_ is enhanced through boron doping.

### 2.5 Hysteresis and Stability

**Figure 5:**
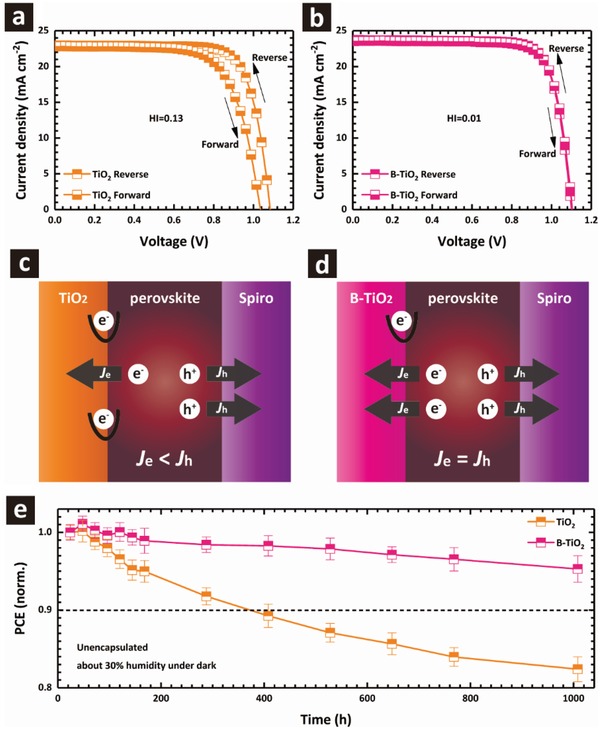
The *J*–*V* curves of the devices with a) TiO_2_ and b) B‐TiO_2_ measured under both reverse‐ and forward‐scan directions. Carrier transport mechanism of mesoscopic‐type PSCs with c) TiO_2_ and d) B‐TiO_2_ ETLs. e) Dark storage stability of unsealed MAPbI_3_ cells based on TiO_2_ and B‐TiO_2_ ETLs in dry air (20 to 25 °C; relative humidity of 25–30%). Error bars represent standard deviations of 12 individual cells for each case.

Hysteresis and stability are two pivotal characteristics for the PSCs.^32^ For the hysteresis behavior, **Figure** **5**a,b shows the *J*–*V* curves measured under both reverse‐ and forward‐scan directions with the related photovoltaic parameters listed in Table 2. It is found that the device with B‐TiO_2_ has almost identical *J*–*V* curves with negligible hysteresis, even when it is measured using different scan rates from 1 to 500 mV s^−1^ (Figure S11, Supporting Information). To quantify the hysteresis effect, hysteric index was given by

(4) $$\mathrm{HI}\;=\;\frac{{\mathrm{PCE}}_{\mathrm{reverse}}-\;{\mathrm{PCE}}_{\mathrm{forward}}}{{\mathrm{PCE}}_{\mathrm{reverse}}}$$

where PCE_reverse_ and PCE_forward_ represent PCE for the reverse (from open‐circuit to short‐circuit under the forward bias voltage) and forward (from short‐circuit to open‐circuit under the forward bias voltage) scans, respectively. PSCs based on B‐TiO_2_ ETL show a decreased HI of 0.01 compared to that of TiO_2_ based analogue at 0.13.

In general, the hysteresis behavior of PSCs is attributed to interfacial capacitance caused by interfacial charge accumulation, which originates from ion migration, high trap density, and unbalanced carrier transport within the device.[qv: 4a–c,33] It is shown that the trap density at the B‐TiO_2_/perovskite interface, one of the major causes for hysteresis, is significantly reduced. Furthermore, the electron mobility of the TiO_2_ ETL is only 3.30 × 10^−5^ cm^2^ V^−1^ s^−1^ (Figure 2c), about an order of magnitude slower than the hole mobility of the doped spiro‐OMeTAD (≈10^−4^ cm^2^ V^−1^ s^−1^) HTL.^34^ Therefore, the electron flux (*J*_e_) is smaller than the hole flux (*J*_h_), which leads to charge accumulation at the TiO_2_/perovskite interface, as shown in Figure 5c. The accumulated carrier would give rise to hysteresis in the PSCs (Figure 5a). In combination with the high electron mobility of the B‐TiO_2_ (1.69 × 10^−4^ cm^2^ V^−1^ s^−1^) ETL and the larger contact area at the ETL/perovskite interface, the *J*_e_ and *J*_h_ can be balanced when the mesoscopic electron conductor (B‐TiO_2_) is employed as the ETL (Figure 5d), resulting in equivalent carrier transport at both interfaces. Thus, the high electron mobility of B‐TiO_2_ would enhance charge transport from perovskite to B‐TiO_2_ ETL, leading to reduced charge accumulation, and consequently, the devices based on the B‐TiO_2_ ETL exhibit negligible hysteresis.

Furthermore, we examined the stability of PSCs made on TiO_2_ and B‐TiO_2_ ETLs under dark storage. Figure 5e shows normalized PCE measured as a function of storage time. These PSCs had good storage stability, while the devices made on B‐TiO_2_ exhibited enhanced stability relative to TiO_2_. The TiO_2_ based device maintains 82% of the initial efficiency after dark storage in an ambient atmosphere over 1000 h, while the B‐TiO_2_ based analogue retains 95% of the original efficiency under the same storage condition. The stability of PSCs is closely related to the interface contact and interfacial charge recombination.[qv: 9,11a,c] In the present work, all PSCs used the same configuration and materials except for ETLs, therefore, the degradation from other interfaces should be the same for all the PSCs. It is found that the trap states density of B‐TiO_2_ films is decreased and charge recombination at B‐TiO_2_/perovskite interface is retarded in comparison to that of pure TiO_2_. The decreased trap states density of B‐TiO_2_ films and retarded charge recombination can facilitate the interfacial charge transfer between the perovskite and ETL so that charge accumulation and recombination within the devices can be avoided. Overall, the stronger binding at the B‐TiO_2_/perovskite interface and the suppressed interfacial recombination account for superior stability in mesoscopic‐type PSCs based on B‐TiO_2_.

## 3 Conclusions

In summary, we have demonstrated an effective way to eliminate the hysteresis effect of PSCs resulting from the ETL by manipulating carrier transport properties using mesoporous B‐TiO_2_ as an improved ETL. The incorporation of B dopant in TiO_2_ ETL not only reduces the hysteresis behavior but also improves *V*_oc_, *J*_sc_, FF, and PCE of PSCs. The simultaneous improvements are mainly ascribed to the following two reasons. First, the substitution of titanium by boron species effectively passivates oxygen vacancy defects in TiO_2_ ETL, leading to increased electron mobility and conductivity, therefore greatly facilitating electron transport. Meanwhile, our DFT calculations also show that B doping leads to stronger binding at the ETL/perovskite interface with a total binding energy of −5.41 eV for TiO_2_/perovskite interface and −6.63 eV for B‐TiO_2_/perovskite interface, due to diminished trap states. This enhanced interfacial binding ensures superior electronic contact between the perovskite and ETL. Second, B dopant upshifts the conduction band edge of TiO_2_, resulting in more efficient electron extraction with suppressed charge recombination. As a result, PSCs based on B‐TiO_2_ ETL demonstrate a higher efficiency of 20.51% than 19.06% of the device with pure TiO_2_ ETL, and the hysteresis is reduced from 0.13% to 0.01% with the B‐TiO_2_ based device showing negligible hysteresis behavior.

## 4 Experimental Section

*Preparation of TiO_2_ Materials*: 60 mL of titanium (IV) isopropoxide (TTIP) (Sigma‐Aldrich, 97%) was added dropwise to a solution consisting of 140 mL of deionized water and 60 mL of ethanol in a 500 mL beaker under vigorous stirring, and the formed precipitate solution was stirred intensely for 4 h after the drip process was finished. After that, the mixture was filtered with a 250 mL G5 sand core funnel, and the precipitate was washed three times with deionized water during the filtration process. The precipitate was then transferred to a Teflon round container (inside volume, 450 mL) containing 90 g trimethylamine aqueous solution (6.58 wt%), and the solution was stirred for 14 h at a temperature of 80–85 °C to form a translucent sol, after which another 10 g of trimethylamine aqueous solution was added and further stirred for 10 min at room temperature. Subsequently, the resulting sol was transferred into Teflon containers (inside volume, 100 mL) and placed in sealed metal autoclave vessels, which were then placed in an oven for 10 h at 200 °C. The obtained gel was diluted with ethanol and was evaporated on a rotary evaporator to remove most of the water, and the gel was then purified by rinsing with ethanol three times and centrifuged. For the doped samples, 8.04 g boric acid (Sigma‐Aldrich, 99.5%) was dissolved in a mixed solution of deionized water and ethanol, and stirred for 30 min before the addition of TTIP. TiO_2_ pastes were then prepared according to previously reported procedures.[qv: 16a]

*Fabrication of Perovskite Devices*: All devices were prepared in ambient air. The TiO_2_ compact (cp‐TiO_2_) layer was prepared by a spray pyrolysis method: a solution containing 0.4 mL of acetylacetone and 0.6 mL of TTIP in 7 mL of isopropanol was sprayed on cleaned and patterned FTO (Pilkington, 15 Ω sq^−1^) substrates at 460 °C using dry air as a carrier gas. Then, the mesoporous TiO_2_ (mp‐TiO_2_) and B‐doped TiO_2_ films were spin‐coated onto the FTO/cp‐TiO_2_ substrate using as‐prepared pastes and calcined at 510 °C for 30 min in air to remove organic components. The perovskite layer was deposited from a precursor solution containing 1.2 m PbI_2_ (Alfa, 99.999%) and 1.2 × 0.98 m CH_3_NH_3_I (Dyesol, 99.9%) in a mixed solvent of DMF and DMSO, in which the volume ratio of DMF to DMSO was 1:4. The perovskite precursor solution was spin‐coated on FTO/cp‐TiO_2_/mp‐TiO_2_ substrate by a consecutive two‐step spin‐coating program at 1100 and 4200 rpm for 15 and 30 s, respectively. During the second step, 110 µL of chlorobenzene was poured onto the spinning substrate 10 s prior the end of the program. The films were then annealed on a hotplate at 100 °C for 15 min. Once cooled down to room temperature, Spiro‐OMeTAD (Xi'an Polymer Light Technology Corp.) was deposited on top of the perovskite layer by spin coating at 4500 rpm for 20 s. The Spiro‐OMeTAD solution was prepared by dissolving 73.53 mg of Spiro‐OMeTAD in 1 mL chlorobenzene, with the addition of 29.30 µL of 4‐tert‐butylpyridine (tBP, Sigma‐Aldrich, 96%), 17.22 µL of lithium bis(trifluoromethanesulfonyl)imide (Li‐TFSI, Sigma‐Aldrich, 99.8%) solution (500 mg Li‐TFSI in 1 mL acetonitrile), and 6.78 µL of tris(2‐(1*H*‐pyrazol‐1‐yl)‐4‐tert‐butylpyridine)‐cobalt(III) tris(bis(trifluoromethylsulfonyl)imide) (FK209, Dyenamo AB) solution (400 mg FK209 in 1 mL acetonitrile).^35^ Finally, an 80 nm gold layer was thermally evaporated on top of the device.

*Characterization*: The morphology measurement was taken by SEM (SU8010, Hitachi, Japan) and TEM (Tecnai G2 F30, FEI, USA). The composition and crystal structure were analyzed by XRD (D8 FOCUS, Bruker, Germany). XPS and UPS measurements were performed on the ESCALAB 250Xi (Thermo Scientific) with different radiation source. The film roughness was collected on an atomic force microscope (5500, Keysight, USA). The contact angle measurement was carried out by a contact angle meter (JC200D3, Powereach, China). The UV–vis absorption spectra were recorded with a spectrophotometer (UV‐3600Plus, Shimadzu, Japan). Steady PL was obtained from a fluorescence spectrometer (FLS980, Edinburgh, UK) with the pump light wavelength of 485 nm and the probe light wavelength of 770 nm.

*J*–*V* curves were recorded by using a Keithley 2420 source meter under simulated sunlight from Newport solar simulator matching the AM 1.5G standard with an intensity of 100 mW cm^−2^. A nonreflective metal mask with an aperture area of 0.09 cm^2^ was used to define the active area of the device. The IPCE curves were confirmed as a function of wavelength from 300 to 800 nm (PV Measurements, Inc.). The IS measurement was carried out on an electrochemical workstation (Zennium pro, Zahner, Germany) in the frequency range of 10 mHz to 1 MHz under AM 1.5G condition.

## Conflict of Interest

The authors declare no conflict of interest.

## Supporting information

Supplementary
