# Supplementary material for: Enhanced Interfacial Binding and Electron Extraction Using Boron‐Doped TiO2 for Highly Efficient Hysteresis‐Free Perovskite Solar Cells
Source: Adv Sci (Weinh). 2019 Sep 10;6(21):1901213. doi: 10.1002/advs.201901213 (PMC6839631; doi:10.1002/advs.201901213)
Supplement: Supplementary file 1 — Supplementary [file ADVS-6-1901213-s001.pdf]

Copyright WILEY-VCH Verlag GmbH & Co. KGaA, 69469 Weinheim, Germany, 2019.

Supporting Information

for *Adv. Sci.,* DOI: 10.1002/advs.201901213

Enhanced Interfacial Binding and Electron Extraction Using Boron-Doped TiO_2 for Highly Efficient Hysteresis-Free Perovskite Solar Cells

Xiaoqiang Shi, Yong Ding, Shijie Zhou, Bing Zhang, Molang Cai, Jianxi Yao, Linhua Hu, Jihuai Wu, Songyuan Dai,* and Mohammad Khaja Nazeeruddin**

Supporting information

Enhanced Interfacial Binding and Electron Extraction Using Boron-Doped TiO_2 for Highly Efficient Hysteresis-Free Perovskite Solar Cells

Xiaoqiang Shi, Yong Ding, Shijie Zhou, Bing Zhang, Molang Cai, Jianxi Yao, Linhua Hu, Jihuai Wu, Songyuan Dai* and Mohammad Khaja Nazeeruddin**

Dr. X. Shi, Dr. Y. Ding, Dr. S. Zhou, Dr. B. Zhang, Dr. M. Cai, Prof. J. Yao, Prof. S. Dai
State Key Laboratory of Alternate Electrical Power System with Renewable Energy Sources, North China Electric Power University, Beijing 102206, P. R. China.
E-mail: sydai@ncepu.edu.cn

Prof. L. Hu, Prof. S. Dai
Key Laboratory of Photovoltaic and Energy Conservation Materials, Institute of Applied Technology, Hefei Institutes of Physical Science, Chinese Academy of Sciences, Hefei, Anhui, 230031, P. R. China.
E-mail: lhhu@rntek.cas.cn

Prof. J. Wu
Fujian Provincial Key Laboratory of Photoelectric Functional Materials, Institute of Materials Physical Chemistry, Huaqiao University, Xiamen 361021, P. R. China.

Prof. M. K. Nazeeruddin
Group for Molecular Engineering of Functional Materials, Institute of Chemical Sciences and Engineering, École Polytechnique Fédérale de Lausanne, CH-1951 Sion, Switzerland.
E-mail: mdkhaja.nazeeruddin@epfl.ch

Keywords: perovskite solar cells, charge transport, titanium dioxide, hysteresis, interfacial binding

Note 1

***Electron mobility of TiO_2 and B-TiO_2*:** Electron-only devices (FTO/cp-TiO_2/TiO_2 or B-TiO_2/PCBM/Ag) were fabricated to calculate the electron mobility of the samples by the SCLC model. The PCBM solution was prepared in chlorobenzene with a concentration of 20 mg/mL, and spin-coated on mesoporous TiO_2 surface at 3000 rpm (20 s), then annealed at 100 °C for 15 min. Sequentially, 60 nm thick Ag was deposited with a shadow mask. The dark *J-V* characteristics of the electron-only devices were measured by a Keithley 2420 source. The mobility is extracted by fitting the *J-V* curves with the Mott-Gurney law [1]

$$J = \frac{9}{8}\varepsilon_0\varepsilon_r\mu_e\frac{\left(V_{app}\text{-}V_r\text{-}V_{bi}\right)^2}{L^3} \qquad \text{(S1)}$$

Where J is the current density, ε_0 is the vacuum permittivity (8.854 × 10^{-12} F/m), ε_r is the dielectric permittivity of the TiO_2 (55) [2], L is the film thickness (180 nm), V_{app} is the applied voltage of the device, V_r is the voltage drop due to constant resistance and series resistance across the electrodes, V_{bi} is the built-in voltage due to the different work function of the two electrodes, and μ_e is the electron mobility. The μ_e is calculated from the currents in the square law region. The electron mobility of 3.30 × 10^{-5} and 1.69 × 10^{-4} cm^2 V^{-1} s^{-1} for pure TiO_2 and B-TiO_2 are calculated from the currents in the square law region, respectively.

Note 2

Details for DFT calculations: the computational calculations were performed by using the Forcite and Vienna ab-initio simulation package (VASP). The geometry optimization calculations were performed using the Forcite module of Materials Studio [3]. The energy and force convergence were set to be 10^{-5} kcal/mol and 10^{-5} kcal/mol/Å. Then the adsorption energy was calculated by VASP [4]. The generalized-gradient approximation (GGA)-Perdew-Burke-Ernzerh (PBE) functional of augmented plane wave was applied. A cutoff energy of 500 eV for the plane-wave basis set and a Monkhorst-Pack mesh of 2 × 3 × 1 for the Brillouin

zone integration are employed. A Vacuum layer of 10 Å is adopted in the calculation. The binding energy $E_{binding}$ between TiO_2 and $MAPbI_3$ is defined as $E_{binding}$ = $E_{(MAPbI3 + TiO2)}$ - $E_{(MAPbI3)}$ - $E_{(TiO2)}$, where $E_{(MAPbI3 + TiO2)}$ is the total energy of $MAPbI_3$ with absorbed TiO_2 and $E_{(MAPbI3)}$ and $E_{(TiO2)}$ are the total energies of pristine $MAPbI_3$ and isolated TiO_2, respectively. In our model, the perovskite models were deposite on the anatase TiO_2 made by 72 TiO_2 units. After optimizing, the Ti-I lengths are 2.64 Å and 2.66 Å. The binding energy is calculated to be -5.41 eV, the negative adsorption energy indicated that the adsorption process is exothermic. When we replaced a Ti by B, the binding energy is calculated to be -6.63 eV, which shows that doping B into TiO_2 is beneficial for the connection between $MAPbI_3$ and TiO_2.

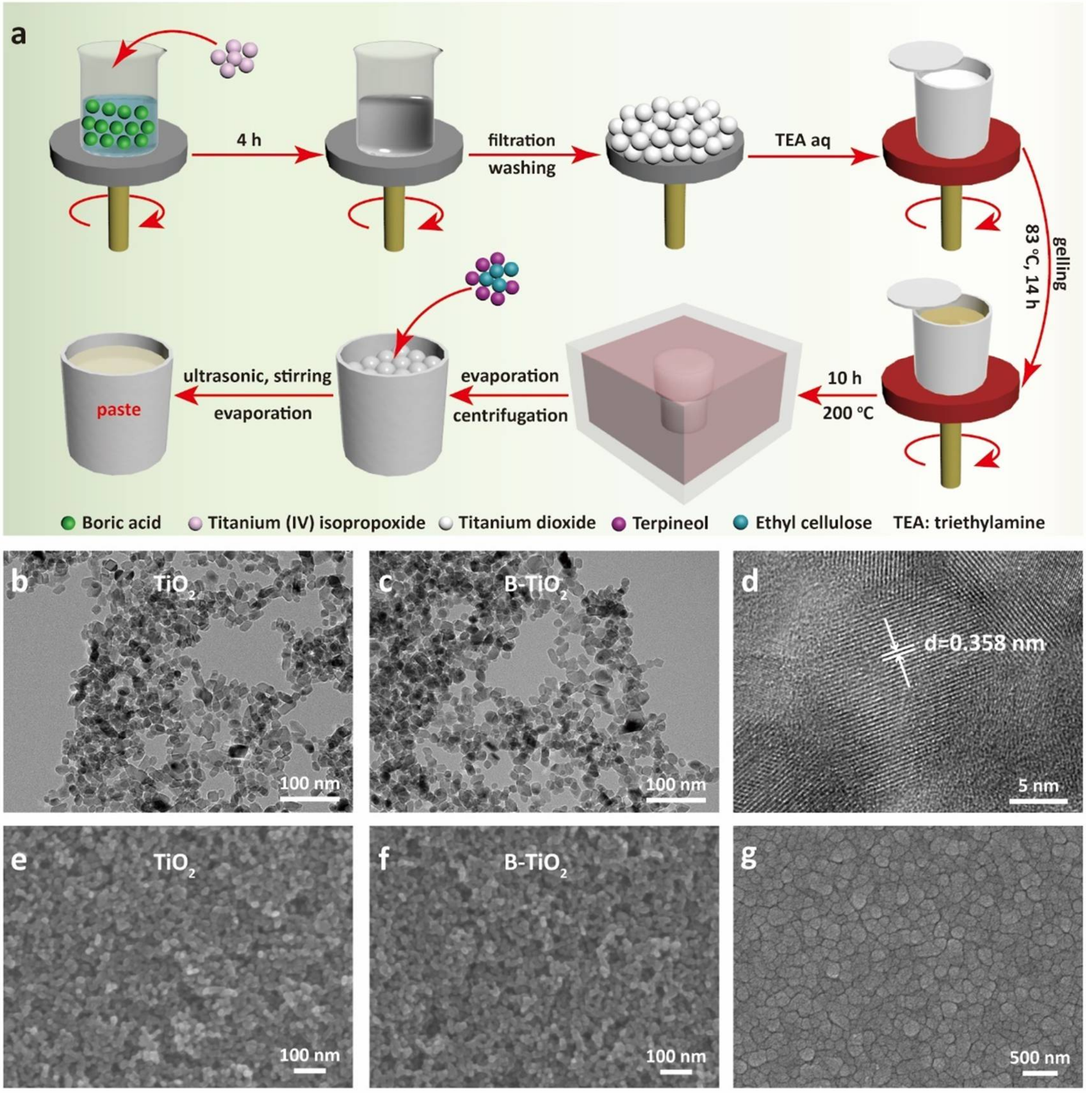

Figure S1. (a) Schematic illustration of the sol-gel process to prepare TiO_2 and B-TiO_2 nanoparticles. (b, e) TEM and SEM images of TiO_2 nanoparticles. (c, d, f) TEM, HRTEM, and SEM images of B-TiO_2 nanoparticles. (g) SEM image of TiO_2 compact layer. TiO_2 and B-TiO_2 films were deposited on identical TiO_2 compact layer (denoted by cp-TiO_2) by spin coating.

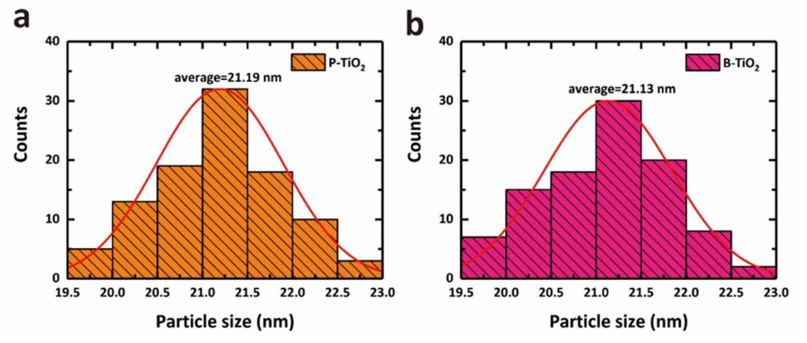

Figure S2. Histograms of the particle size distributions obtained from TEM images for (a) TiO_2 and (b) B-TiO_2, obtained from analysis of 100 particles per sample.

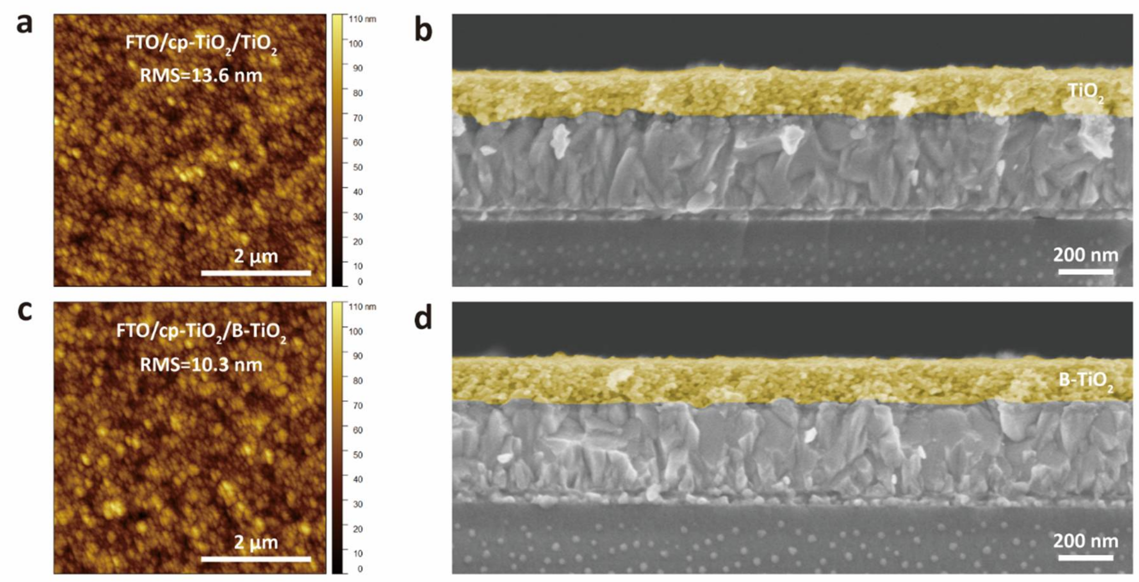

Figure S3. AFM images and corresponding cross-section views of (a, b) TiO_2 and (c, d) B-TiO_2 films.

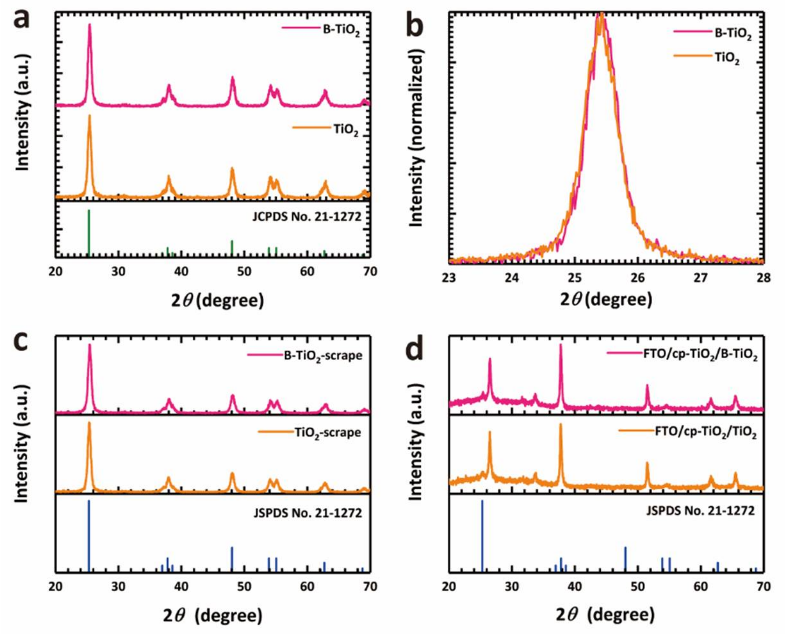

Figure S4. (a) XRD patterns of TiO_2 and B-TiO_2 powder. (b) Zoomed-in XRD patterns of TiO_2 and B-TiO_2 powder for the (101) plane. XRD patterns of (c) TiO_2 and B-TiO_2 powder scraped from corresponding sintered films and (d) TiO_2 and B-TiO_2 films deposited on FTO/cp-TiO_2.

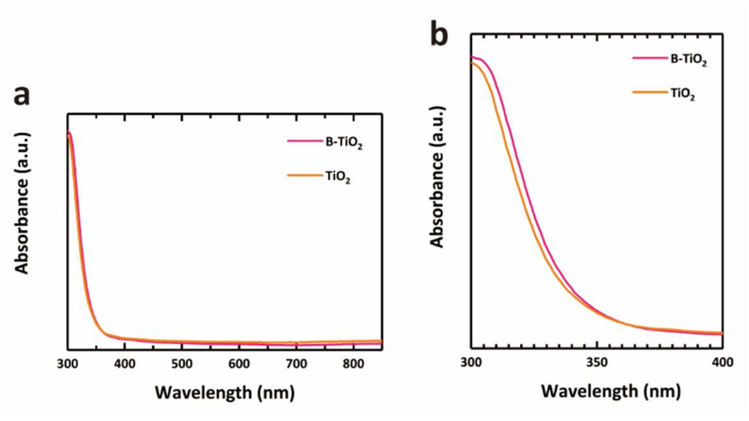

Figure S5. (a) UV-vis absorption spectra of TiO_2 and B-TiO_2. (b) Details of the UV-vis absorption spectra of TiO_2 and B-TiO_2 in the 300-400 nm range.

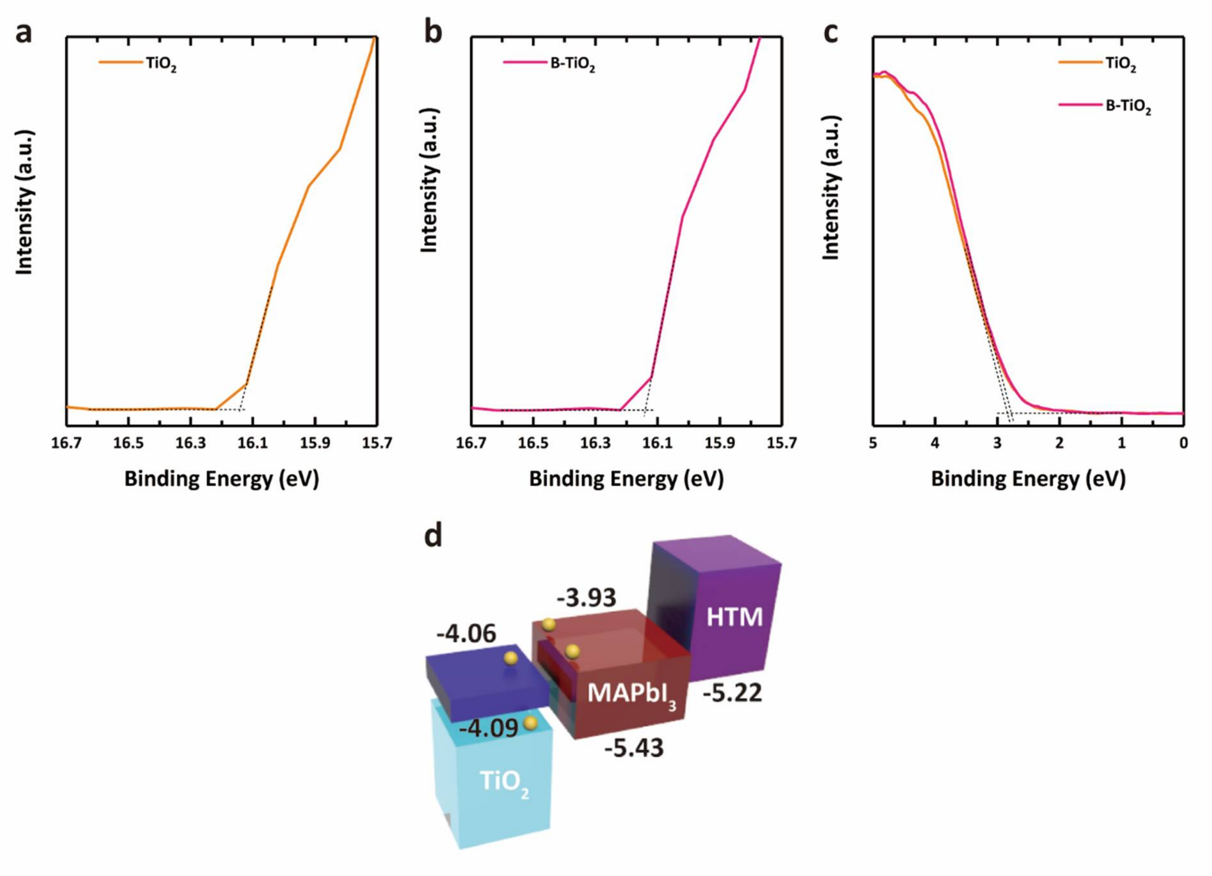

Figure S6. (a) Ultraviolet photoelectron spectroscopy (UPS) secondary electron cutoff of TiO_2. (b) UPS cutoff edge of B-TiO_2. (c) Valence band spectra of TiO_2 and B-TiO_2. (d) Diagram of energy levels (relative to the vacuum level) of functional layers in the device.

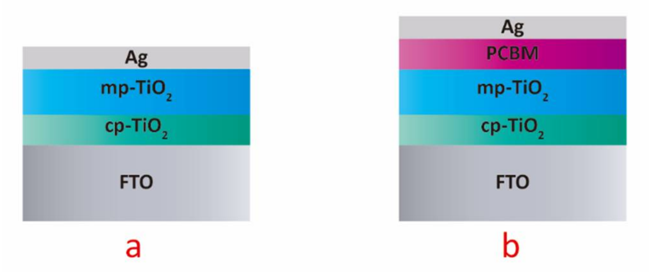

Figure S7. Device structures for electrical measurements. (a) FTO/cp-TiO_2/mp-TiO_2/Ag for conductivity and trap-state density measurement. (b) FTO/cp-TiO_2/mp-TiO_2/PCBM/Ag for mobility measurement.

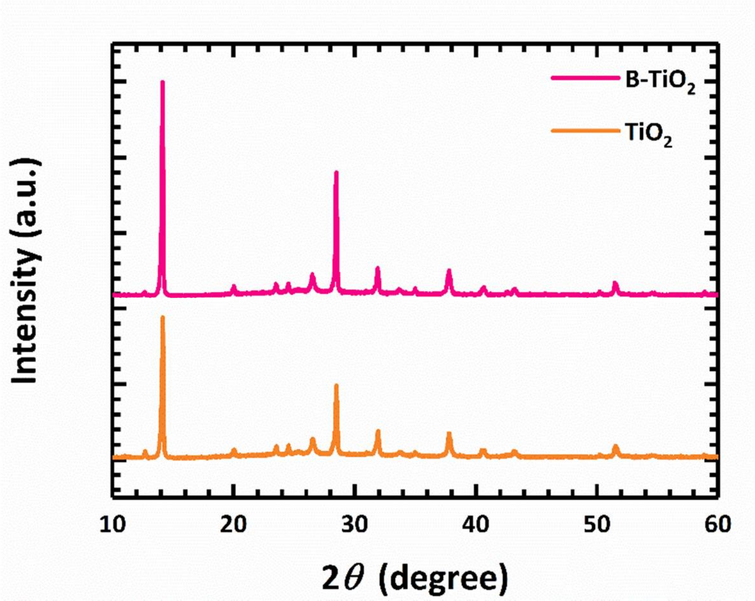

Figure S8. XRD patterns of perovskite films deposited on the TiO_2 and B-TiO_2 substrates.

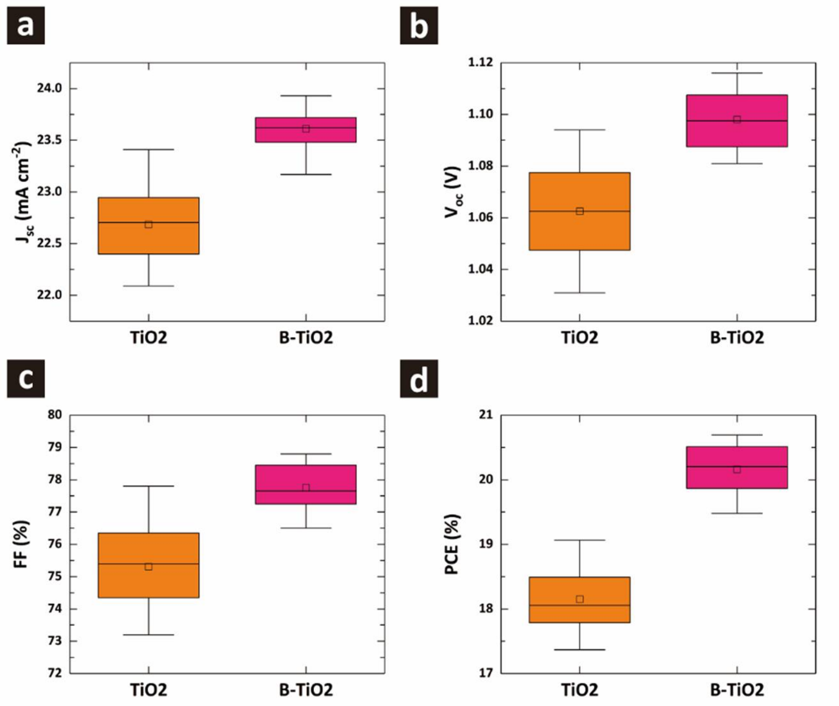

Figure S9. Statistics of the (a) J_{sc}, (b) V_{oc}, (c) FF, and (d) PCE of the PSCs based on TiO_2 and B-TiO_2 ETL. The short horizontal lines above and below the box indicate the maximum and minimum values, respectively. The three horizontal lines in the box show the 25th, 50th and 75th percentiles of the values for the devices. The hollow squares represent the average values.

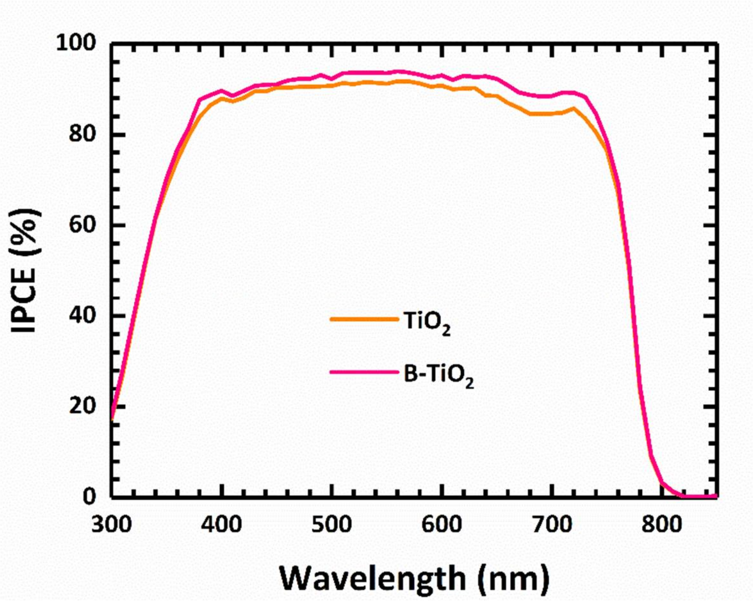

Figure S10. Incident photon to current conversion efficiency (IPCE) curves of TiO_2 and B-TiO_2 based devices.

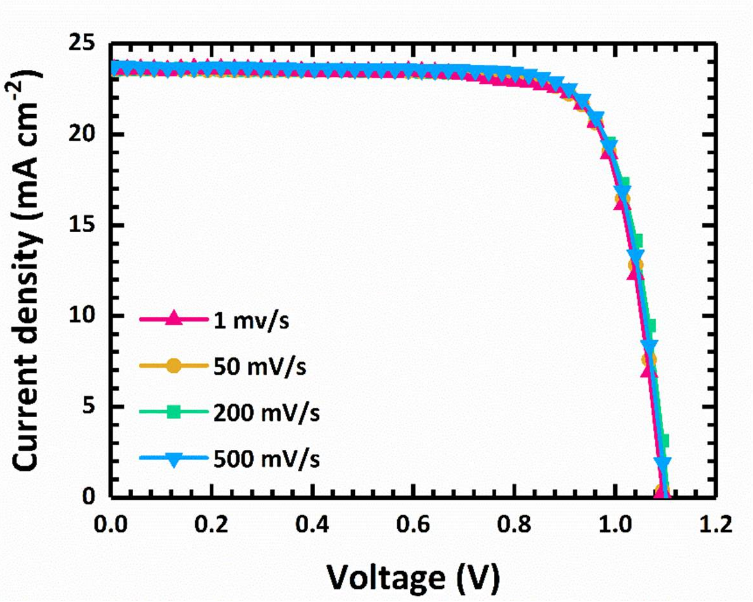

Figure S11. *J*-*V* curves of the PSCs using B-TiO_2 ETL measured under different scanning rates.

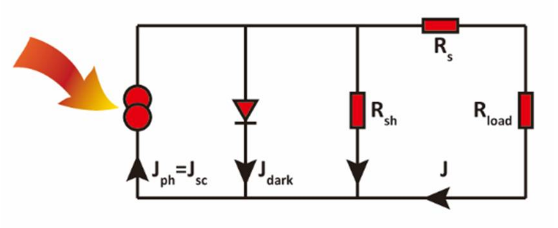

Fig S12. Equivalent circuit of solar cells including series and shunt resistances.

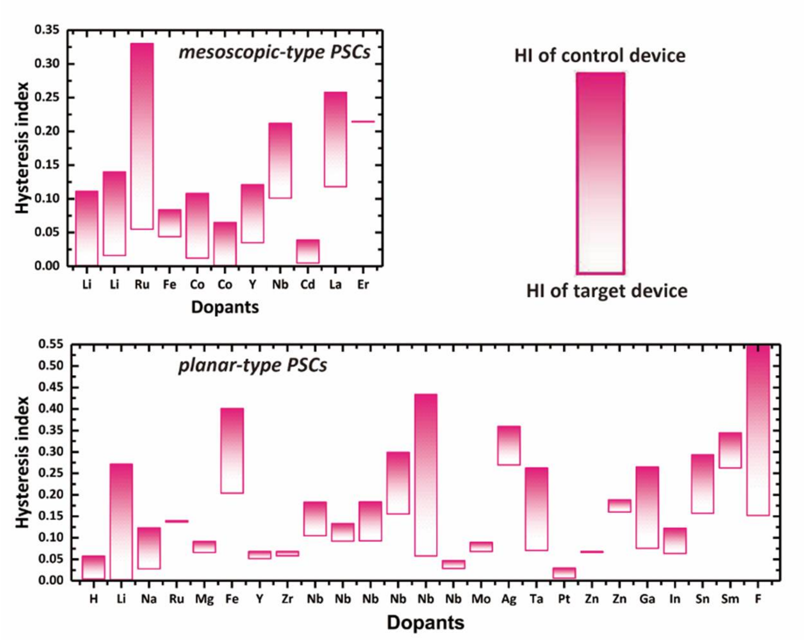

Figure S13. Comparison of the hysteresis index (HI) of PSCs comprised of doped TiO_2 electron transport layer. The references are listed in Table S2.

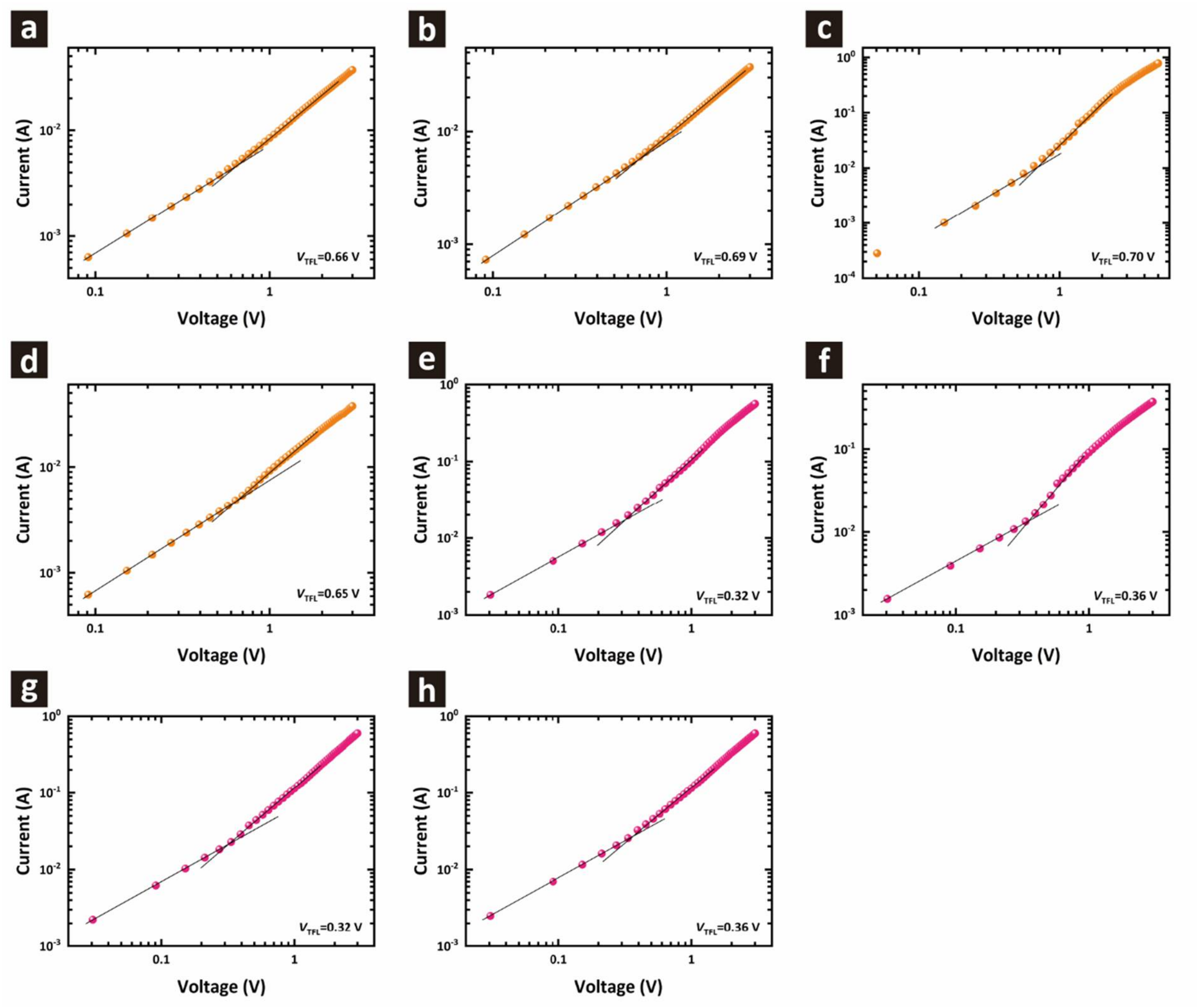

Figure S14. Log-log plot of *I-V* curves of TiO_2 (a-d) and B-TiO_2 (e-h) devices revealing V_{TFL} tuning point behavior. The averaged V_{TFL} value was calculated to be 0.675 and 0.34 for TiO_2 and B-TiO_2, respectively, which were used to determine the trap state densities.

Table S1. Conductivity of TiO_2 and B-TiO_2 films.

Films	σ_0/mS cm^{-1}	μ_e/cm^2 V^{-1} s^{-1}	n_t/cm^{-3}
TiO_2	6.00×10^{-3}	3.30×10^{-5}	1.33×10^{17}
B-TiO_2	1.81×10^{-2}	1.69×10^{-4}	6.01×10^{16}

Table S2a. Summary of the doped TiO_2 films for mesoscopic-type PSCs.

Ref.	Film	Dopant	$Control_{rev}$	$Control_{for}$	$Control_{HI}$	$Target_{rev}$	$Target_{for}$	$Target_{HI}$

[5]	Li-doped mp-TiO_2	Li	15.71	13.97	0.111	17.27	17.25	0.001
[6]	Li-doped mp-TiO_2	Li	17.10	14.70	0.140	19.30	19.00	0.016
[7]	Ru-doped cp-TiO_2+mp-TiO_2	Ru	14.83	9.94	0.330	18.35	17.34	0.055
[8]	Fe-doped cp-TiO_2+mp-TiO_2	Fe	14.59	13.37	0.084	18.01	17.22	0.044
[9]	Co-doped mp-TiO_2	Co	14.92	13.31	0.108	18.16	17.94	0.012
[10]	Co-doped mp-TiO_2	Co	18.28	17.10	0.065	19.60	19.58	0.001
[11]	Y-doped mp-TiO_2	Y	17.50	15.38	0.121	19.99	19.29	0.035
[12]	Nb-doped mp-TiO_2	Nb	17.00	13.40	0.212	19.80	17.80	0.101
[13]	Cd-doped mp-TiO_2	Cd	6.63	6.37	0.039	8.30	8.26	0.005
[14]	La-doped mp-TiO_2	La	12.11	8.98	0.258	15.42	13.06	0.118
[15]	Er-doped mp-TiO_2	Er	9.64	7.58	0.214	14.06	11.04	0.215

cp=compact, mp=mesoporous, rev=reverse, for=forward, HI= hysteresis index.

Table S2b. Summary of the doped TiO_2 films for planar-type PSCs.

Ref.	Film	Dopant	Control$_{rev}$	Control$_{for}$	Control$_{HI}$	Target$_{rev}$	Target$_{for}$	Target$_{HI}$
[16]	Hydrogenated cp-TiO_2	H	16.58	15.63	0.057	19.30	19.22	0.004
[17]	Li-doped cp-TiO2	Li	16.21	16.17	0.002	13.22	9.63	0.272
[18]	Na-doped cp-TiO2	Na	15.40	13.50	0.123	18.00	17.50	0.028
[19]	Ru-doped cp-TiO_2	Ru	13.42	11.58	0.137	15.70	13.50	0.140
[20]	Mg-doped cp-TiO_2	Mg	13.56	12.32	0.091	15.73	14.69	0.066
[21]	Fe-doped cp-TiO_2	Fe	11.05	6.62	0.401	14.70	11.70	0.204
[22]	Y-doped cp-TiO_2	Y	16.59	15.46	0.068	15.46	14.66	0.052
[22]	Zr-doped cp-TiO_2	Zr	16.59	15.46	0.068	15.69	14.78	0.058
[23]	Nb-doped cp-TiO_2	Nb	13.10	10.70	0.183	15.20	13.60	0.105
[24]	Nb-doped cp-TiO_2	Nb	16.34	14.16	0.133	19.27	17.49	0.092
[25]	Nb-doped cp-TiO_2	Nb	13.66	11.15	0.184	14.29	12.96	0.093
[26]	Nb-doped cp-TiO_2	Nb	18.14	12.72	0.299	19.20	16.21	0.156
[27]	Nb-doped cp-TiO_2	Nb	12.82	7.26	0.434	16.12	15.19	0.058

[28]	Nb-doped cp-TiO_2	Nb	19.10	18.20	0.047	20.80	20.20	0.029
[22]	Mo-doped cp-TiO_2	Mo	16.59	15.46	0.068	15.21	13.85	0.089
[29]	Ag-doped cp-TiO_2	Ag	11.70	7.50	0.359	14.10	10.30	0.270
[30]	Ta-doped cp-TiO_2	Ta	6.63	4.89	0.262	9.94	9.24	0.070
[31]	Pt-doped cp-TiO_2	Pt	16.72	16.23	0.029	19.15	19.03	0.006
[22]	Zn-doped cp-TiO_2	Zn	16.59	15.46	0.068	15.91	14.86	0.066
[32]	Zn-doped cp-TiO_2	Zn	10.60	8.90	0.160	13.80	11.20	0.188
[33]	Ga-doped cp-TiO_2	Ga	15.41	11.33	0.265	16.89	15.61	0.076
[34]	In-doped cp-TiO_2	In	16.30	14.30	0.123	18.90	17.70	0.063
[35]	Sn-doped cp-TiO_2	Sn	13.30	9.40	0.293	17.20	14.50	0.157
[36]	Sm-doped cp-TiO_2	Sm	12.78	8.38	0.344	14.10	10.40	0.262
[37]	F-doped cp-TiO_2	F	8.80	4.00	0.545	15.80	13.40	0.152

cp=compact, rev=reverse, for=forward, HI= hysteresis index.

References

[1] A. M. Goodman, A. Rose, *J. Appl. Phys.* **1971**, 42, 2823.

[2] L. Kavan, N. Tétreault, T. Moehl, M. Grätzel, *J. Phys. Chem. C* **2014**, 118, 16408.

[3] K.-S. Chang, T. Yoshioka, M. Kanezashi, T. Tsuru, K.-L. Tung, *Chem. Commun.* **2010**, 46, 9140.

[4] a) G. Kresse, J. Furthmüller, *Comput. Mater. Sci.* **1996**, 6, 15; b) G. Kresse, D. Joubert, *Phys. Rev. B* **1999**, 59, 1758.

[5] J. H. Heo, M. S. You, M. H. Chang, W. Yin, T. K. Ahn, S.-J. Lee, S.-J. Sung, D. H. Kim, S. H. Im, *Nano Energy* **2015**, 15, 530.

[6] F. Giordano, A. Abate, J. P. Correa Baena, M. Saliba, T. Matsui, S. H. Im, S. M. Zakeeruddin, M. K. Nazeeruddin, A. Hagfeldt, M. Graetzel, *Nat. Commun.* **2016**, 7, 10379.

[7] S. Wang, B. Liu, Y. Zhu, Z. Ma, B. Liu, X. Miao, R. Ma, C. Wang, *Solar Energy* **2018**,

169, 335.

[8] X. Gu, Y. Wang, T. Zhang, D. Liu, R. Zhang, P. Zhang, J. Wu, Z. D. Chen, S. Li, *J. Mater. Chem. C* **2017**, 5, 10754.

[9] S. Sidhik, A. Cerdan Pasarán, D. Esparza, T. López Luke, R. Carriles, E. De la Rosa, *ACS Appl. Mater. Interfaces* **2018**, 10, 3571.

[10] J. K. Kim, S. U. Chai, Y. Ji, B. Levy-Wendt, S. H. Kim, Y. Yi, T. F. Heinz, J. K. Nørskov, J. H. Park, X. Zheng, *Adv. Energy Mater.* **2018**, 8, 1801717.

[11] M. Li, Y. Huan, X. Yan, Z. Kang, Y. Guo, Y. Li, X. Liao, R. Zhang, Y. Zhang, *ChemSusChem* **2018**, 11, 171.

[12] Y. Numata, R. Ishikawa, Y. Sanehira, A. Kogo, H. Shirai, T. Miyasaka, *J. Mater. Chem. A* **2018**, 6, 9583.

[13] Y. Li, Y. Guo, Y. Li, X. Zhou, *Electrochimica Acta* **2016**, 200, 29.

[14] X.-X. Gao, Q.-Q. Ge, D.-J. Xue, J. Ding, J.-Y. Ma, Y.-X. Chen, B. Zhang, Y. Feng, L.-J. Wan, J.-S. Hu, *Nanoscale* **2016**, 8, 16881.

[15] Z. Ren, J. Wu, N. Wang, X. Li, *J. Mater. Chem. A* **2018**, 6, 15348.

[16] X. Yao, J. Liang, Y. Li, J. Luo, B. Shi, C. Wei, D. Zhang, B. Li, Y. Ding, Y. Zhao, X. Zhang, *Adv. Sci.* **2017**, 4, 1700008.

[17] D. Liu, S. Li, P. Zhang, Y. Wang, R. Zhang, H. Sarvari, F. Wang, J. Wu, Z. Wang, Z. D. Chen, *Nano Energy* **2017**, 31, 462.

[18] J. Ma, X. Guo, L. Zhou, Z. Lin, C. Zhang, Z. Yang, G. Lu, J. Chang, Y. Hao, *ACS Appl. Energy Mater.* **2018**, 1, 3826.

[19] Z. Xu, X. Yin, Y. Guo, Y. Pu, M. He, *J. Mater. Chem. C* **2018**, 6, 4746.

[20] X. Liu, Z. Liu, B. Sun, X. Tan, H. Ye, Y. Tu, T. Shi, Z. Tang, G. Liao, *Electrochimica Acta* **2018**, 283, 1115.

[21] X. Li, F. Hao, X. Zhao, X. Yin, Z. Yao, Y. Guo, H. Shen, H. Lin, *ACS Appl. Mater. Interfaces* **2017**, 9, 34833.

[22] H.-H. Wang, Q. Chen, H. Zhou, L. Song, Z. S. Louis, N. D. Marco, Y. Fang, P. Sun, T.-B. Song, H. Chen, Y. Yang, *J. Mater. Chem. A* **2015**, 3, 9108.

[23] B.-X. Chen, H.-S. Rao, W.-G. Li, Y.-F. Xu, H.-Y. Chen, D.-B. Kuang, C.-Y. Su, *J. Mater. Chem. A* **2016**, 4, 5647.

[24] I. Jeong, H. Jung, M. Park, J. S. Park, H. J. Son, J. Joo, J. Lee, M. J. Ko, *Nano Energy* **2016**, 28, 380.

[25] J. Song, S. P. Li, Y. L. Zhao, J. Yuan, Y. Zhu, Y. Fang, L. Zhu, X. Q. Gu, Y. H. Qiang, *J. Alloy. Compd.* **2017**, 694, 1232.

[26] G. Yin, J. Ma, H. Jiang, J. Li, D. Yang, F. Gao, J. Zeng, Z. Liu, S. F. Liu, *ACS Appl. Mater. Interfaces* **2017**, 9, 10752.

[27] C. Liang, P. Li, Y. Zhang, H. Gu, Q. Cai, X. Liu, J. Wang, H. Wen, G. Shao, *J. Power Sources* **2017**, 372, 235.

[28] Y. Lv, B. Cai, Q. Ma, Z. Wang, J. Liu, W.-H. Zhang, *RSC Adv.* **2018**, 8, 20982.

[29] M.-C. Wu, Y.-H. Liao, S.-H. Chan, C.-F. Lu, W.-F. Su, *Solar RRL* **2018**, 2, 1800072.

[30] R. Ranjan, A. Prakash, A. Singh, A. Singh, A. Garg, R. K. Gupta, *J. Mater. Chem. A* **2018**, 6, 1037.

[31] L.-L. Jiang, Z.-K. Wang, M. Li, C.-H. Li, P.-F. Fang, L.-S. Liao, *Solar RRL* **2018**, 1800149.

[32] M.-C. Wu, S.-H. Chan, M.-H. Jao, W.-F. Su, *Sol. Energy Mater. Sol. Cells* **2016**, 157, 447.

[33] H. Liu, Z. Zhang, X. Zhang, Y. Cai, Y. Zhou, Q. Qin, X. Lu, X. Gao, L. Shui, S. Wu, J.-M. Liu, *Electrochimica Acta* **2018**, 272, 68.

[34] J. Peng, T. Duong, X. Zhou, H. Shen, Y. Wu, H. K. Mulmudi, Y. Wan, D. Zhong, J. Li, T. Tsuzuki, K. J. Weber, K. R. Catchpole, T. P. White, *Adv. Energy Mater.* **2017**, 7, 1601768.

[35] Q. Cai, Y. Zhang, C. Liang, P. Li, H. Gu, X. Liu, J. Wang, Z. Shentu, J. Fan, G. Shao,

Electrochimica Acta **2018**, 261, 227.

[36] Y. Xiang, Z. Ma, J. Zhuang, H. Lu, C. Jia, J. Luo, H. Li, X. Cheng, *J. Phys. Chem. C* **2017**, 121, 20150.

[37] V. Zardetto, F. di Giacomo, H. Lifka, M. A. Verheijen, C. H. L. Weijtens, L. E. Black, S. Veenstra, W. M. M. Kessels, R. Andriessen, M. Creatore, *Adv. Mater. Interfaces* **2018**, 5, 1701456.
